# Supplementary material for: Transcriptomic Profiling Reveals an Enhancer RNA Signature for Recurrence Prediction in Colorectal Cancer
Source: Genes (Basel). 2023 Jan 3;14(1):137. doi: 10.3390/genes14010137 (PMC9859145; doi:10.3390/genes14010137)
Supplement: Supplementary file 1 [file genes-14-00137-s001.zip › Additional file 1.pdf]

Transcriptomic profiling reveals an enhancer RNA signature for recurrence prediction in colorectal cancer

Divya Sahu[1,2], Chen-Ching Lin[3], Ajay Goel[1,*]

[1]Department of Molecular Diagnostics and Experimental Therapeutics, Beckman Research Institute of City of Hope Comprehensive Center, 1218 S. Fifth Avenue, Biomedical Research Center, Monrovia, California 91016, USA

[2]Department of Genetics, University of Alabama at Birmingham, Birmingham, AL, 35294, USA

[3]Institute of Biomedical Informatics, National Yang Ming Chiao Tung University, Taipei, 11221, Taiwan

***Correspondence:** ajgoel@coh.org; Tel.: +1-626-218-3452

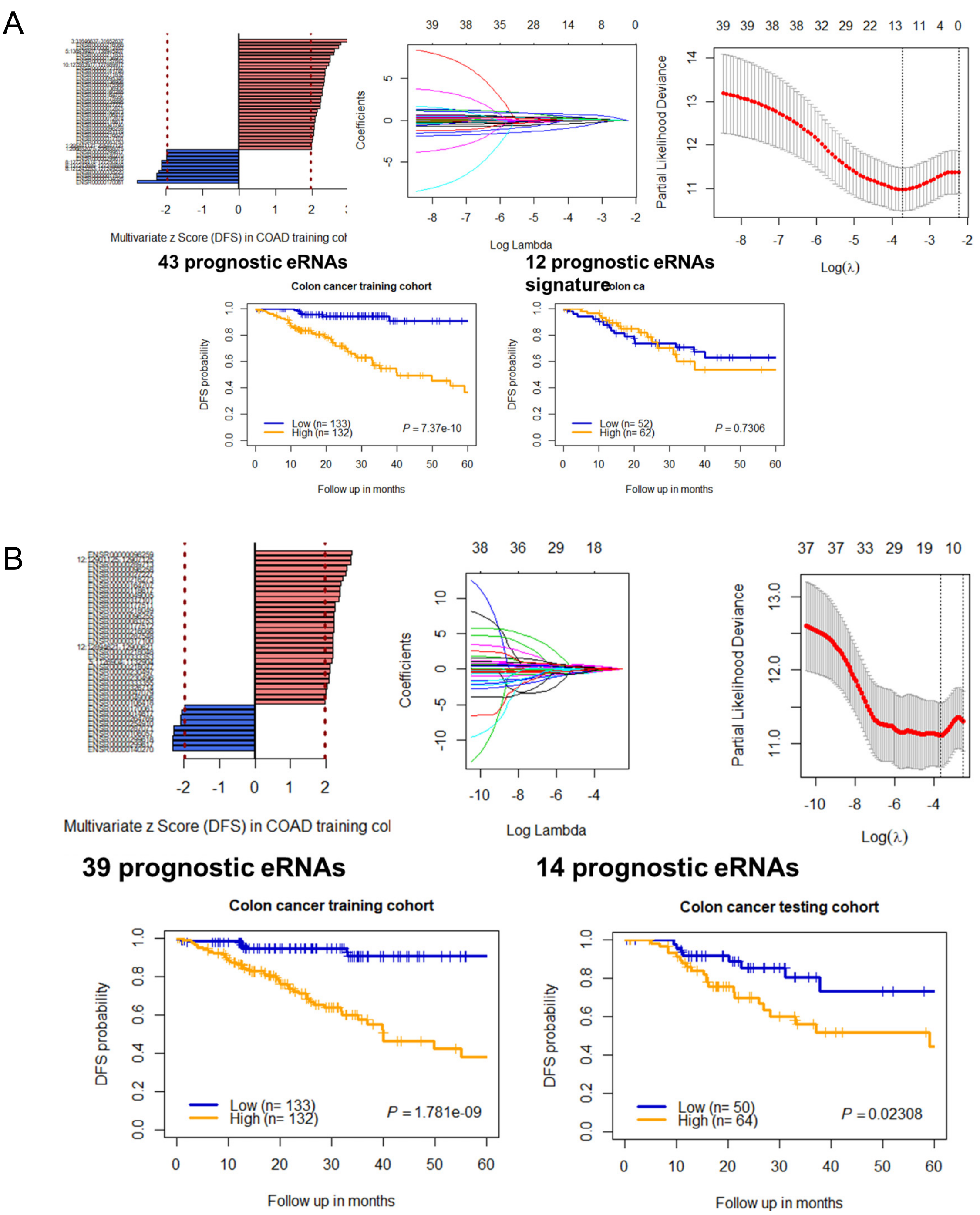

Figure S1: Demonstration of identification of prognostics eRNA signatures in failed model and successful model in the testing set. **(A)** In one of the trial bar graphs show multivariate cox analysis of eRNAs independently associated with disease-free survival after adjusting with clinical risk factors including patient age, gender and TNM stage. Then Lasso cox regularization was applied on the training set and eRNAs were selected with non-zero coefficient. The selected eRNA was used to build risk score model. Next, Kaplan-Meier plot shows eRNA signature can significantly stratify patients into low and high risk-score groups in training set but not in testing set. Therefore,

it is termed as failed model. **(B)** In another trial eRNA signature significantly stratify patients into two risk-score groups both in training set and in testing set, and therefore it is termed as successful model.
