# Supplementary material for: Transcriptomic Profiling Reveals an Enhancer RNA Signature for Recurrence Prediction in Colorectal Cancer
Source: Genes (Basel). 2023 Jan 3;14(1):137. doi: 10.3390/genes14010137 (PMC9859145; doi:10.3390/genes14010137)
Supplement: Supplementary file 1 [file genes-14-00137-s001.zip › Additional file 2.pdf]

Transcriptomic profiling reveals an enhancer RNA signature for recurrence prediction in colorectal cancer

Divya Sahu[1,2], Chen-Ching Lin[3], Ajay Goel[1,*]

[1]Department of Molecular Diagnostics and Experimental Therapeutics, Beckman Research Institute of City of Hope Comprehensive Center, 1218 S. Fifth Avenue, Biomedical Research Center, Monrovia, California 91016, USA

[2]Department of Genetics, University of Alabama at Birmingham, Birmingham, AL, 35294, USA

[3]Institute of Biomedical Informatics, National Yang Ming Chiao Tung University, Taipei, 11221, Taiwan

***Correspondence:** ajgoel@coh.org; Tel.: +1-626-218-3452

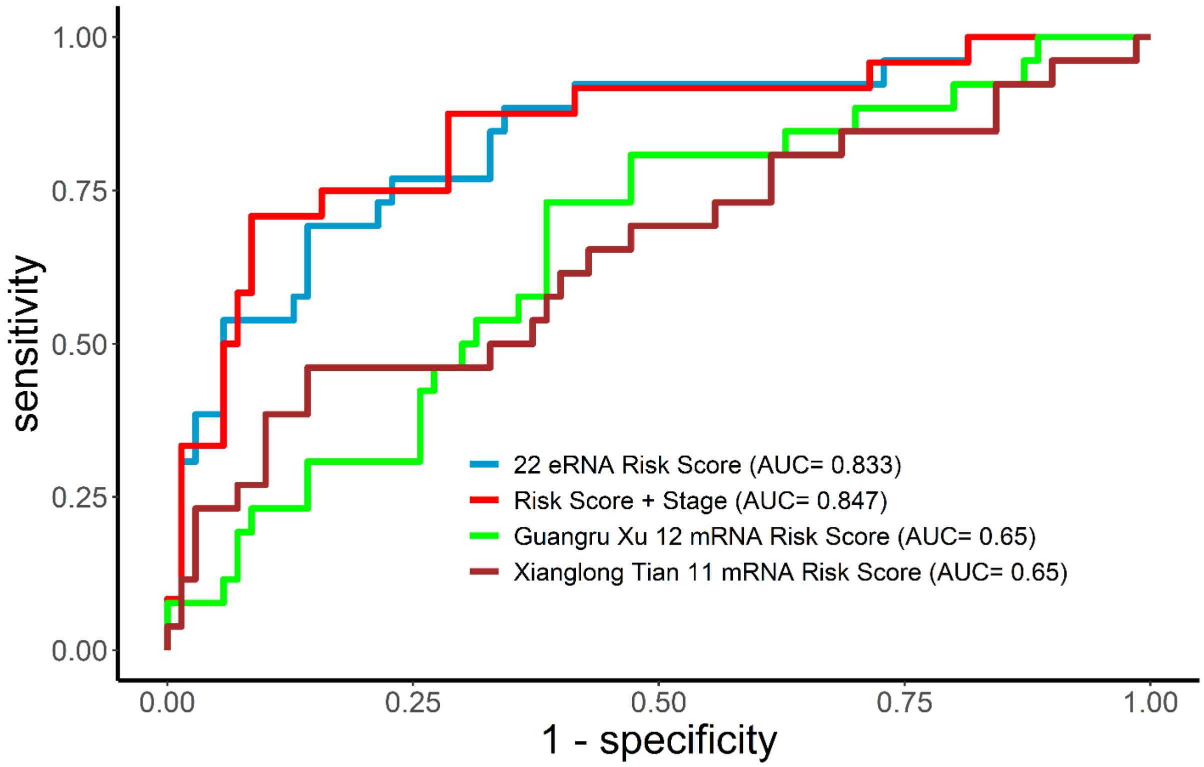

Figure S2: Receiver operating characteristics curve analyses of colon cancer recurrence prediction by the enhancer RNA signature and protein-coding gene signature. AUC=area under the curve.
