# Supplementary material for: Transcriptomic Profiling Reveals an Enhancer RNA Signature for Recurrence Prediction in Colorectal Cancer
Source: Genes (Basel). 2023 Jan 3;14(1):137. doi: 10.3390/genes14010137 (PMC9859145; doi:10.3390/genes14010137)
Supplement: Supplementary file 1 [file genes-14-00137-s001.zip › Additional file 3.pdf]

Transcriptomic profiling reveals an enhancer RNA signature for recurrence prediction in colorectal cancer

Divya Sahu[1,2], Chen-Ching Lin[3], Ajay Goel[1,*]

[1]Department of Molecular Diagnostics and Experimental Therapeutics, Beckman Research Institute of City of Hope Comprehensive Center, 1218 S. Fifth Avenue, Biomedical Research Center, Monrovia, California 91016, USA

[2]Department of Genetics, University of Alabama at Birmingham, Birmingham, AL, 35294, USA

[3]Institute of Biomedical Informatics, National Yang Ming Chiao Tung University, Taipei, 11221, Taiwan

***Correspondence:** ajgoel@coh.org; Tel.: +1-626-218-3452

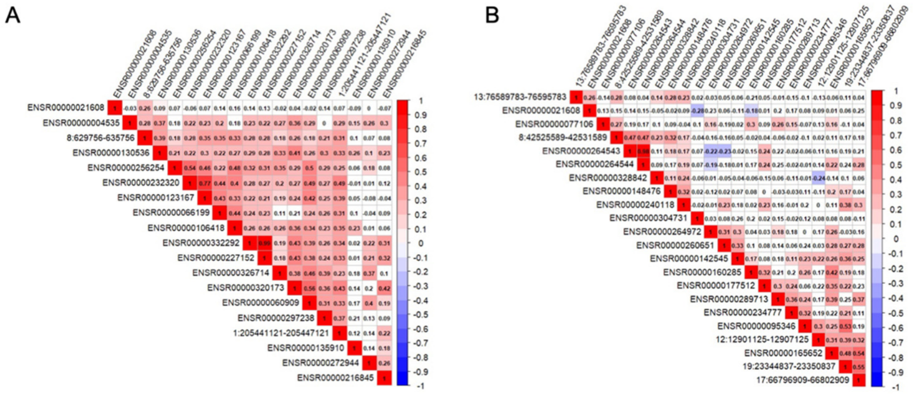

Figure S3: Spearman correlation analysis of the enhancer RNA signature (eRNA). Corrplot of eRNA expression levels measured by RNA-seq in **(A)** colon cancer; 22 eRNAs **(B)** rectal cancer; 19 eRNAs. The scale bar denotes correlation strength, with 1 indicating positive correlation (red) and -1 indicating negative correlation (blue). Only eRNA *ENSR00000021608* was observed in common between colon cancer and rectal cancer.
