# Supplementary material for: Transcriptomic Profiling Reveals an Enhancer RNA Signature for Recurrence Prediction in Colorectal Cancer
Source: Genes (Basel). 2023 Jan 3;14(1):137. doi: 10.3390/genes14010137 (PMC9859145; doi:10.3390/genes14010137)
Supplement: Supplementary file 1 [file genes-14-00137-s001.zip › Additional file 4.pdf]

Transcriptomic profiling reveals an enhancer RNA signature for recurrence prediction in colorectal cancer

Divya Sahu[1,2], Chen-Ching Lin[3], Ajay Goel[1,*]

[1]Department of Molecular Diagnostics and Experimental Therapeutics, Beckman Research Institute of City of Hope Comprehensive Center, 1218 S. Fifth Avenue, Biomedical Research Center, Monrovia, California 91016, USA

[2]Department of Genetics, University of Alabama at Birmingham, Birmingham, AL, 35294, USA

[3]Institute of Biomedical Informatics, National Yang Ming Chiao Tung University, Taipei, 11221, Taiwan

***Correspondence:** ajgoel@coh.org; Tel.: +1-626-218-3452

Table S1: Clinical characteristics of TCGA colorectal cancer patients in the entire cohort, training cohort and testing cohort.

**Subgroup**	**Entire cohort**	**Training cohort**	**Testing cohort**
		**Colon Cancer**	
**Number of patients**	96	48	48
**Age (years)**			
Median	65	66.5	64
Range	(34-90)	(40-86)	(34-90)
**Tumor Stage**			
I and II	52	22	30
III	42	24	18
**Gender**			
Female	43	19	24
Male	53	29	24
		**Rectal Cancer**	
**Number of patients**	121	60	61
**Age (years)**			
Median	64	64.5	64
Range	(31-90)	(33-90)	(31-86)
**Tumor Stage**			
I and II	73	35	38
III	42	22	20
**Gender**			
Female	54	29	25
Male	67	31	36
